# Imaging Patterns in Breast Cancer for Women Under 40 Years: A Descriptive Cohort Study

**DOI:** 10.1007/s44197-023-00169-2

**Published:** 2024-01-11

**Authors:** Amal A. Alhaidary, Ahmad R. Al-Qudimat, Haitham Arabi, Raed M. Al-Zoubi

**Affiliations:** 1 Women’s Imaging, Department of Radiology, King Abdul Aziz Medical City, Riyadh, Saudi Arabia; 2 King Saud Bin Abdulaziz University for Health Sciences, Riyadh, Saudi Arabia; 3 King Abdullah International Medical Research Center, Riyadh, Saudi Arabia; 4 Surgical Research Section, Department of Surgery, Hamad Medical Corporation, Doha, Qatar; 5 Department of Public Health, College of Health Sciences, QU-Health, Qatar University, Doha, Qatar; 6 Department of Pathology, King Abdul Aziz Medical City, Riyadh, Saudi Arabia; 7 Department of Biomedical Sciences, College of Health Sciences, QU-Health, Qatar University, 2713 Doha, Qatar; 8 Department of Chemistry, Jordan University of Science and Technology, P.O. Box 3030, Irbid, 22110 Jordan

**Keywords:** Breast cancer, Young age, Radiology, Pathology, Below 40 years, Retrospective study

## Abstract

**Background and Aim:**

Breast cancer is the most frequently occurring malignant disease in women and remains the leading cause of cancer-related deaths among females worldwide. The aim of this study is to evaluate the imaging findings of breast cancer in women under the age of 40 and analyze their pathological patterns.

**Method:**

A retrospective study was conducted from 2013 to 2019, involving 120 patients below 40 years of age with pathologically confirmed primary epithelial breast cancers. The data were collected from the electronic records of a tertiary hospital in Riyadh, Saudi Arabia. Mammograms were performed for 115 patients, ultrasounds were conducted for all patients, and MRI scans were carried out for 47 patients.

**Results:**

All radiological findings and clinical characteristics of the 120 cases were retrieved from our digital-based system. The majority of breast cancer patients (83.4%) were between 30 and 40 years old, and the most common clinical presentation was a mass (45.8%). Out of the 73 patients who underwent genetic tests, 32.9% tested positive for gene mutations. No statistically significant correlation was found between specific age groups and breast composition (*P* = 0.216), specific mammogram abnormalities such as masses (*P* = 0.262), or microcalcifications (*P* = 0.421). Ultrasonography was performed for all patients, with abnormalities detected in only one patient who was diagnosed with Paget’s disease of the nipple. Masses, with or without parenchymal changes, were the predominant feature in 88.3% of cases.

**Conclusion:**

The imaging findings in breast cancer cases typically involve masses with suspicious features, irregular shape, and spiculated margins on mammograms, and irregular shape with microlobulated or angular margins on ultrasound. MRI features commonly include masses with irregular shape and heterogeneous enhancement. The luminal B subtype was identified as the most prevalent pathological feature, characterized by a high proliferative index (Ki-67%).

**Graphical Abstract:**

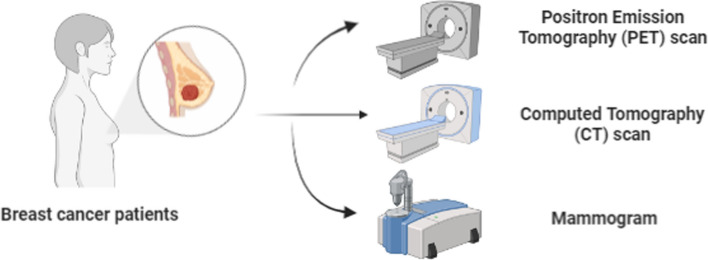

## Introduction

Breast cancer (BC) is the second most common cancer worldwide according to the World Health Organization (WHO) [1]. BC represents the most prevalent malignancy among women and the first cause of death from cancer in females worldwide [2, 3]. There were 2,261,419 new cases of BC worldwide in 2020 (11.7% of all cancers) according to recent data from the Global Cancer Observatory of WHO. BC caused 6.9% of cancer deaths worldwide in 2020 [4]. The incidence and mortality of BC is the highest among other cancers and has been on the rise in recent years, with a higher prevalence in older women [5]. The BC among young adult’s accounts for approximately 5–7% in Western and 13–27% in Asian populations. In Kingdom of Saudi Arabia (KSA), BC accounts for about 24% for patients below 35 years according to Saudi Cancer Registry in 2015 [6–9]. This reflects those various geographic areas differ in their age structure. Although BC in young adults uncommon disease, statistics demonstrated elevated incidence [10].

There are evidence-based predisposing risk factors associated with BC in young adults. For instance, early menarche, which defined as menarche before age of [11], radiation exposure for disease therapy as in lymphoma before age of 20, reproductive factors including oral contraceptives and a family history of BC [11, 12].

To date, no concrete definition of young-age BC with different studies using various ages as a prognostic factor. A wide array of reports has indicated that age is an independent prognostic factor; however, this issue remains controversial since BC among young women is more likely to be associated with more aggressive subtype such as TNBC and is more likely to be presented at an advanced stage due to the complex biology that defines its subtype or due to a low index of suspicion and delayed diagnosis [13]. Thus, loco-regional recurrences and distant metastases are often displayed which contributes to the poor clinical outcomes.

The advancements of breast imaging modalities have crucially contributed to the detection of BC in young ages. Mammogram has its limitation in detection of lesions in young women because of the dense breast tissue which has been associated with younger age and premenopausal status [14, 15]. Therefore, screening using the mammogram alone has no significant benefit in young age population [16]. Additionally, low clinical suspicion and different radiological features of BC in young women contribute to the complexity of the diagnosis. In this context, ultrasound is considered the most useful initial method for diagnosis. Despite being a more sensitive method compared to the mammogram [17–20], a negative result is usually observed due to the presence of microcalcifications that are seen in the mammogram and, therefore, both screenings are crucial in diagnosis [18, 21]. For women with a BRCA gene mutation, the American College of Radiology (ACR) has recommended MRI as an initial screening modality begins at age of 25 then to be performed in addition to mammography annually after the age of 30 [22].

Increasing the knowledge of the imaging features of BC among young adults along with their histopathological characteristics is essential for providing the diagnosis. To the best of our knowledge, there are no previous studies in Saudi Arabia that had studied the BC imaging features with the corresponding pathological profile in women under the age of 40 particularly with age group. Therefore, this study is aimed at providing a better understanding of the association between imaging findings and the pathological features in patients under the age of 40 to provide additional cautions and guidelines in BC screening programs.

## Methods and Materials

Data were collected of all women below 40 with biopsy-proven primary epithelial breast cancer between January 2013 and December 2019 retrospectively and the study was approved by the Institutional Review Board (IRB). We have included all the primary epithelial tumors and excluded those who have incomplete pathological and radiological data and who were treated outside. The records were collected from the electronic database of a tertiary hospital in Riyadh, Saudi Arabia. Clinical data and patient’s demographics including age, gender, clinical presentation, risk factors, type of treatment, pregnancy/ lactation and living status were collected from the hospital clinical information system.

### Pathological Data

The data of 115 cases were collected from the pathological database, including tumor types, hormonal receptor status of estrogen (ER), Progesterone (PR), and Human Epidermal Growth Receptor 2 (HER 2) which were classified according to ASCO 2014 and tumor grade along with proliferative index (Ki-67). Ki-67 was classified according to previous literatures into < 15% and ≥ 15% [23]. Accordingly, molecular subtypes are considered as luminal A (ER + and/or PR + , HER 2 negative and Ki-67 < 15%), luminal B with HER 2 negative (ER + and/or any PR and Ki-67 ≥ 15%), and luminal B with HER 2 + (ER + and/or any PR +) HER 2 type (with the hormonal negative) and triple-negative types (hormonal and HER are negative) [24].

### Radiological Data

The image analysis of every case was reviewed by one radiologist specialized in breast imaging. The mammogram, ultrasound, and the MRI scans were analyzed using the latest American College of Radiology Breast Imaging Reporting and Data System (ACR- BI-RADS) the 5th edition.

Digital mammograms using tomosynthesis examinations in standard views were performed. Mammography was performed on 115/120 patients while the remaining 5 were in a medical situation prevented them from doing mammogram. The analysis included breast composition, mass shape, margin, density, location, and presence of suspicious microcalcifications as well as additional findings, if present, as asymmetries, architectural distortion, and skin changes. Breast ultrasound for the 120 cases was performed by skilled sonographers using high-resolution linear transducers in B-mode and color flow, and the findings were analyzed retrospectively. The masses were described according to the shape, margin, echogenicity, orientation, posterior features, size, and location. Two additional terms were not included in the ACR criteria which are parenchymal changes in case there is no mass to describe and the desmoplastic reaction.

MRI was performed for 47 cases to evaluate the extent of the disease and to evaluate the other side, using 1.5 and 3 Tesla machines with standard hospital protocol including DWI and ADC maps. The masses were analyzed for the shape, margins, pattern of enhancement, type of kinetic curve in which type I is a progressive and type II is a plateau and type III is a washout, and Signal intensity in T2WI. The non-mass enhancement (NME) was described according to the pattern of enhancement and distribution. The results of CT scan staging in non-pregnant women along with chest x-ray and abdominal ultrasound in pregnant women were collected from the reports in the PACS system.

### Statistical Data

All data were collected using Microsoft Excel and analyzed using the statistical program SAS (version 9.4). Chi and Fisher’s Exact tests were used to perform the correlation between categorical variables and the association was considered significant when the *P* value was < 0.05. The median of the patients’ age and tumor size were also calculated.

## Results

### Clinical Data

All radiological data were retrieved from our digital-based system. Clinical characteristics are presented in Table 1. Among 120 cases, only 61 cases have documentations about the initial presentation and 95.3% among these were symptomatic especially a palpable lump and 3 with metastasis upon screening. Additionally, 46.7% were more than 35 of age and 15.8% pregnant during the study. During the initial staging, 24.2% with metastasis mainly to the liver. One case only displayed skin changes (0.8%). There were 73 of patients with genetic tests in which 32.9% has positive gene mutation.

**Table 1:** Initial characteristics of 120 patients

Characteristics	Number (%)
Age	120
20–24	3 (2.5)
25–29	17 (14.2)
30–34	44 (36.7)
35–40	56 (46.7)
Clinical presentation	64
Mass	55 (45.8)
Nipple discharge	3 (2.5)
Mass and nipple discharge	1 (0.8)
Metastasis	3 (2.5)
Skin changes	1 (0.8)
Pain	1 (0.8)
Genetic	73
BRCA 1	9 (12.3)
BRCA 2	14 (19.2)
Both	1 (1.461)
Negative	49 (67.1)

**Fig. 1:**
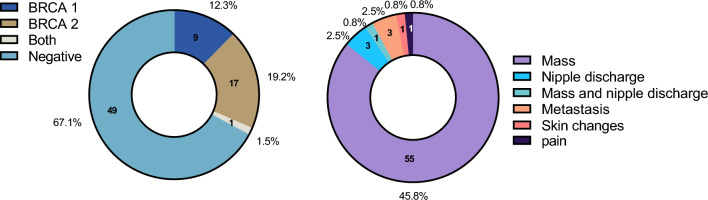
Age groups, genetics and Initial Clinical presentations of 120 BC patients enrolled in this study

The majority of BC patients (83.4%) were aged between 30 and 40 years old, and mass was the most displayed clinical presentation (45.8%) among 120 patients as shown in Fig. 1.

### Imaging Findings

The radiological findings of tumors are summarized in Table 2. There were 100 /120 (83.3%) of patients diagnosed initially in our institute, in which 79.0% were categorized BIRADS 4C and BIRADS 5, and 11.0% were coded BIRADS 4A for which 7 cases of BIRADS A were found below 35 age old. The mean size of the tumors was 36.3 mm. About 84/120 (70.0%) of patients had a biopsy-proven axillary lymph node metastasis. Skin changes were present in 34.2% patients, and multifocal/multi-centric tumors were found in 29.2%.

**Table 2:** Radiological findings

Mammogram features		Ultrasound features		MRI features	
Characteristics	Number (%)	Characteristics	Number (%)	Characteristics	Number (%)
Breast composition		US background		MRI findings	
Fatty	11 (9.2)	Multi-centric	21 (17.5)	Minimal	11 (23.4)
Scattered	35 (29.2)	Multifocal	14 (11.8)	Mild	18 (38.3)
Heterogeneous dense	48 (40.0)	Single	84 (70.6)	Moderate	15 (31.9)
Extremely dense	21 (17.5)			Marked	3 (6.4)
Mass density		Mass shape		MRI mass enhancement	
Equal	11 (13.4)	Oval	31 (29.8)	Homogenous	1 (2.5)
High	71 (86.6)	Round	2 (1.9)	Heterogenous	35 (87.5)
		Irregular	71 (68.3)	Rim	4 (10)
Mass shape		Mass margin		T2 signal intensity	
Oval	16 (13.9)	Circumcribed	9 (8.7)	Hypointense	12 (25.5)
Round	19 (16.5)	Microlobulated and angulated	44 (42.3)	Isointense	24 (51.1)
Irregular	47 (40.9)	Indistinct	22 (21.1)	Hyperintense	11 (23.4)
Mass margin		Spiculated	29 (27.9)	Kinetic curve	
Circumcribed	10 (8.7)	Echogenicity		Type I	1 (2.13)
Obscured	7 (6.1)	Isoechoic	1 (0.9)	Type II	10 (21.3)
Microlobulated	6 (5.2)	Hypoechoic	65 (61.3)	Type I	36 (76.6)
Indistinct	13 (11.3)	Complex	8 (7.6)	Non-mass distribution	
Spiculated	46 (40.0)	Heterogeneous	32 (30.2)	Linear	1(6.7)
Microcalcifications				Segmental	10 (66.7)
Amorphous	2 (3.9)			Regional	2 (13.3)
Punctate	1 (2)			Diffuse	2 (13.3)
Pleomorphic	42 (82.3)			Non-mass enhancement	
Coarse heterogeneous	4 (7.8)			Homogenous	1(6.7)
				Heterogenous	5 (33.3)
Linear branching	2 (3.9)			Clumped	6 (40)
				Clustered rings	3 (20)

**Fig. 2:**
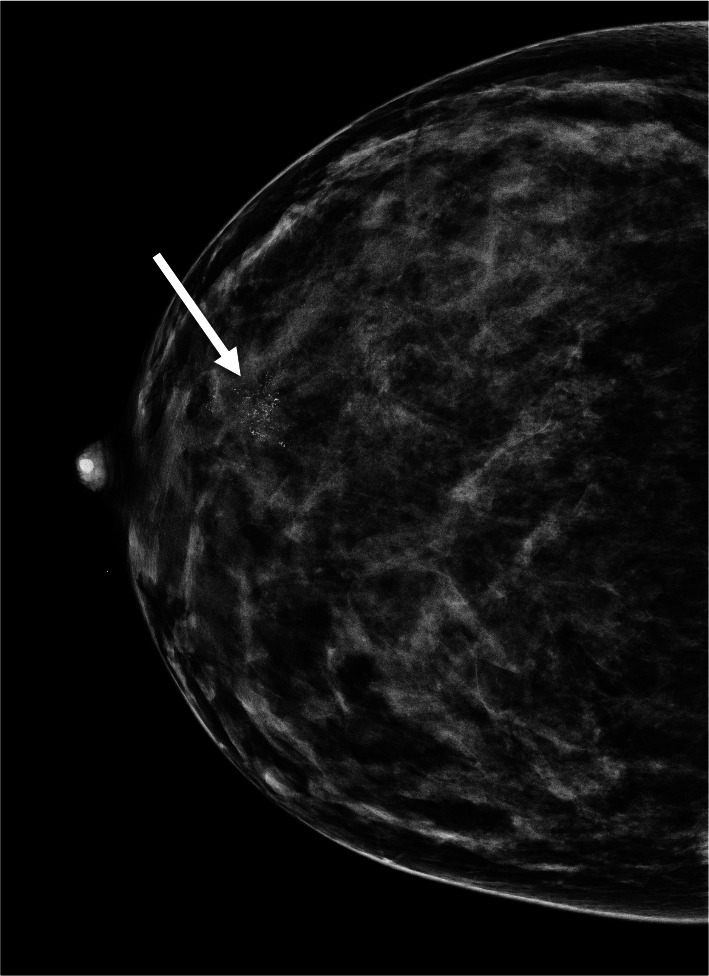
A mammogram of a 37-year-old female with suspicious microcalcifications (arrow) at retroareolar region in dense breast

There was no mammographic abnormality noted for 2 cases. The most common compositions of the breasts were heterogeneous density in 48/115 (40.0%). The mammographic finding was a mass with or without microcalcifications 82/115 (71.3%), followed by suspicious microcalcifications with or without a mass 50/115 (43.5%) (Fig. 2). No statistically significant correlation to specific age group was found whether related to breast composition (*P* = 0.216) or to specific mammogram abnormality in terms of masses (*P* = 0.262) or in microcalcifications (*P* = 0.421).

Ultrasonography was applied for all patients. There was no abnormality seen except in one patient which was diagnosed as Paget’s disease of the nipple. Masses were the dominant feature with or without parenchymal changes in (88.3%). We have noticed that the parenchymal changes, as the only sonographic findings, were not visualized for patients under-age of 30. Additionally, the large masses above 5 cm were noticed in the age group of 30–34 and this age group showed more death compared to other groups.

Among 47 cases who underwent MRI, 40 patients (85.2%) demonstrated enhanced mass with or without non-mass enhancement, and 7 patients (14.9%) had shown a non-mass enhancement only. Most of the patients 76.6% (36 patients) had a type 3 (washout) pattern of enhancement by dynamic curve. In T2W signal intensity, 51.1% (24 patients) were found predominantly intermediate signal intensity.

### Histopathological Data

The most common histological type was found to be invasive ductal carcinoma with or without DCIS (85%) and Grade 3 was seen in 63.3% (Table 3). With regards to subtypes, there is no statistically correlation was found (*P* = 0.247) between a particular molecular subtype and a specific age group.

**Table 3:** Pathological findings

Histopathology	Number (%)
Type	
IDC (NOS)	62 (51.7)
IDC + DCIS	39 (32.5)
DCIS	12 (10)
ILC	1 (0.8)
ILC + IDC	1 (0.8)
Metaplastic	4 (3.3)
Tumor grade	
Grade I	4 (3.3)
Grade II	31 (25.8)
Grade III	76 (63.3)
Ki-67%	
< or equal 15%	17 (14.2)
> 15%	92 (76.7)
HER 2	
Positive	59 (55.1)
Negative	48 (44.9)

## Discussion

The present study was designed to determine the imaging features of BC in young women under the age of 40 in addition to the pathological nature of the disease. The BC in this age group is presented with aggressive histopathological features but it is uncommon. Most of the cases were symptomatic and were presenting with a palpable lump (45.8%) even with the presence of positive family history for BC (11.3%), which is in line with other studies [9, 21, 25, 26]. This could be explained by the decreased awareness for screening in those who have a family history of BC or because of fears. In addition, the majority was sporadic in nature and only 18.4% had a genetic-related BRCA mutation similar to another study [13].

In general, BC in young women is presented in advanced stages [27]. The results of this study demonstrated that around 70% of the cases presented with lymph node involvement and 24.2% had distant metastasis at the time of diagnosis.

The high amount of the fibro-glandular tissue results in dense mammograms composition may affect the detection of the disease [14, 15]. Moreover, the mammograms were found abnormal in 95.7% despite dense breast, which was presented in more than half of the cases and consistent with the literature [10, 22, 26]. Additionally, it BIRADS 5 was confidently reported in 75.0%. This reflects the importance of mammogram in detecting lesions providing a crucial tool in the diagnosis. Interestingly, we found that in the extremely dense breasts suspicious microcalcifications in most of the cases had shown and therefore, the lesions were detected.

**Fig. 3:**
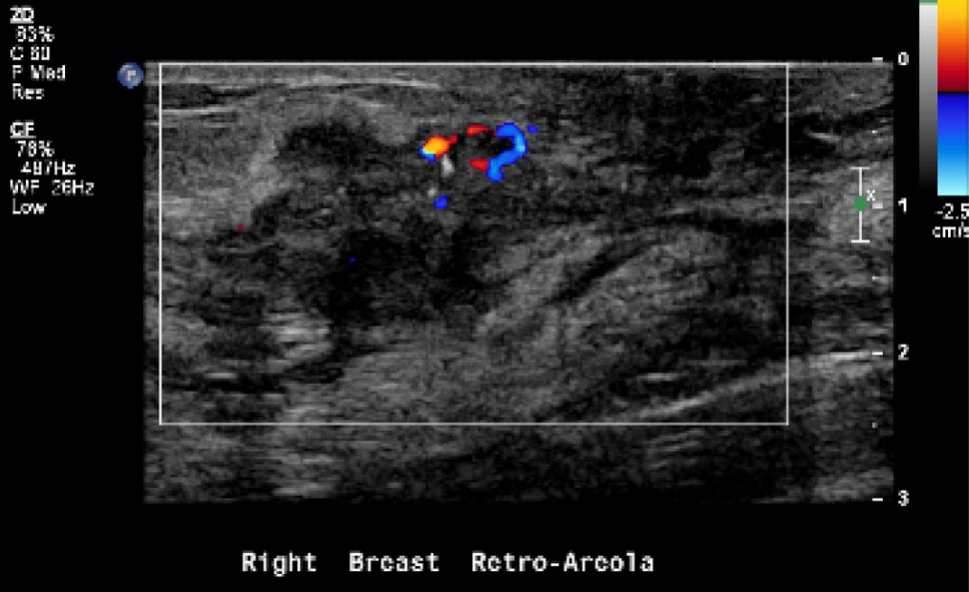
The ultrasound for the same patient shows an irregular-shaped mass with calcifications at retroareolar region

**Fig. 4:**
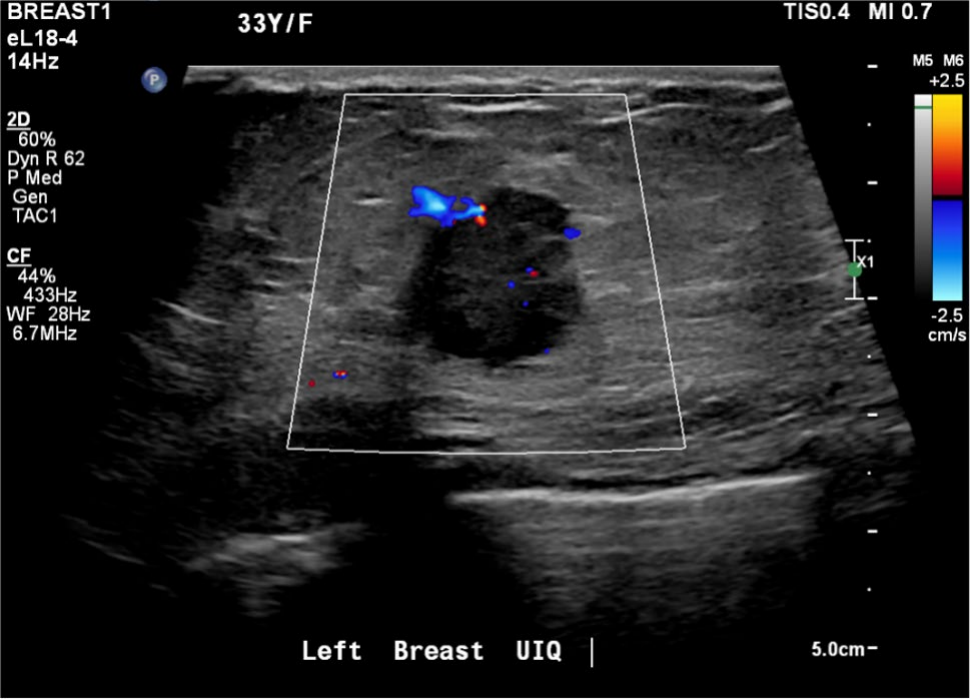
An ultrasound for a 33-year old female shows a round mass with angular margins

The most important mammographic relevant finding was mass alone (43.5%) followed by mass with microcalcifications (26.1%) consistent with previous observations [18, 22, 24, 28]. The masses were frequently irregular in shape (40.9%) and about 40.0% have spiculated margins, which is concordant to other studies [10, 20]. On the other hand, these findings are contrary to An et al. study which had suggested that the masses are more with indistinct margins, and a possible explanation for this might be due to the selectivity of age group, in which more than 30 years of age were omitted in their study [23]. In terms of age group in this study, 83.3% of the cases were 30 years old and above, in which 46.7% of them are representing more than 34 years. Ultrasound is the commonest modality used to diagnose young women due to the absence of radiation [24, 26]. Accordingly, most of the abnormal findings (81.7%) were related to masses in which 44.5% of them were ≥ 5 cm. The masses were mainly irregular in shape (68.3%) (Fig. 3) and showed microlobulated or angular margins (42.3%) (Fig. 4). Similar results were reported by Bullier et al. in which masses had spiculated margins in 27.88% [20]. We found that ultrasound has detected about 99.2% of abnormalities, in which the only one negative case was related to Paget’s disease of the nipple with no associated mass and found also negative in the mammogram. There are several possible explanations for the high predictive value of ultrasound and due to multiple factors: Firstly, is the presence of masses as the main findings. Secondly, is the expertise of radiologists and technologists. Lastly, are due to the existence of high resolutions machines.

Most of the studies have shown that the imaging features of masses in young are similar to benign lesions in terms of circumscribed margins, oval shape and posterior acoustic features. In this study, there were about 6 oval-shaped masses coded below BIRADS 4B and the biopsies were done because of either their margins were relatively non-circumscribed, or their echotextures were complex. An et al. reported a study and recommended that careful examination of the margins is crucial in oval-shaped masses as that can decrease the misinterpretation of the masses as benign lesions [23]. In addition, the majority MRI findings of BC patients (84.9%) had no posterior features, which was similar to some other studies [21, 23].

Most of our cases had mild background parenchymal enhancement post-contrast administration by MRI. Out of 46 cases, 38 cases presented with masses only (68.1%). We found that most of the masses were irregular in shape (70.0%), had irregular margins (45.0%), heterogeneous enhancement (87.5%), and showed a washout pattern of enhancement, which is in line with the literature showing that young breast cancer mainly presented with mass in MRI [20, 24, 28].

Regarding the NME, they frequently had a segmental distribution (66.7%) and had clumped type of enhancement (40.0%). It has been suggested that clumped enhancement is associated with lymph node involvement [28]. This does not appear to be the case in our study, and this might be due to the few numbers of cases that have been examined by the MRI. With respect to the histopathological analysis, it is not surprising that most of the cases were infiltrating ductal carcinoma and only two cases [1.7%] were infiltrative lobular carcinoma (ILC). We showed that most of the tumors (63.3%) were high grade with a high proliferative index (Ki-67) in 76.7% which matched those observed in earlier studies [10, 11, 22, 27, 29].

Different researchers have shown different results regarding the molecular subtype in young women affected with BC which likely reflects the geographic variation [25, 30, 31]. The current study found that luminal B (46.9%) is the commonest subtype similar to other studies [10, 20, 26, 32], followed by HER-2 positive (26.1%). Additionally, luminal A and TNBC in this age group were infrequent and account for 22.6% and 7.0% respectively which was consistent with previous data [12, 33]. Around 30% of the cases of BC in young women had metastasis at the time of our study period. During the initial staging, about 24.2% of patients had metastasis and 18.18% who were free of the disease initially then had metastasis during the period of treatment.

With regards to the surgical management, most of the women below the age of 40 who have BC underwent mastectomy more than conservative therapy similarly to our results in which 50.0% underwent mastectomies [33, 34].

Although the risk of death in a young patient below the age of 40 is high even when diagnosed in the early stages compared to advanced stages in patient more than 40 years [26, 28], we found that most of the patients who died due to BC during the period of the study presented initially in an advanced stage with metastasis. Moreover, the death was high among patients less than 35 and occurred within the first 4 years.

Since there is about a quarter of BC affecting women at a young age in our population and around half of the cases are more than 35 years, we recommend to further investigations about the benefit of routine breast ultrasound after 35 years old in our population for those who have no family history and before going into the screening program.

### Limitation

Although this study highlights the importance of BC investigation among young women, there are some limitations were posed by the study. Due to the retrospective nature of the study in which the data were digitally extracted from the system, some of the data were missed due to part of the treatment or the investigations were undertaken elsewhere. Additionally, the sample size was small that might affect the results. Finally, the examinations were operator dependent, and the given images were analyzed from the PACS according to the latest ACR-BIRADS criteria, but the report’s BIRADS maintained as was written to avoid bias caused by the known diagnosis during the analysis.

## Conclusion

Breast cancer among young women below the age of 40 in our study population usually presented symptomatically with a large lump with lymph nodes involvement regardless of positive family history which may reflect the decrease of awareness of the disease or sensation of fear. Since about a quarter of BC patients in our population are from young women, and around half of the cases are more than 35 years in this study, we recommend doing further investigations in the feasibility of routine breast ultrasound after 35 years old for those who have no family history before going into the screening program. The imaging findings are usually worrisome for malignancy, the masses have irregular shape and spiculated margins in mammogram and have irregular shape with Microlobulated /angular margins in ultrasound. The MRI features are usually of mass with irregular shape and heterogeneous enhancement. Their pathology is common of luminal B subtype of high grade and high proliferative index (Ki-67%).

## Data Availability

All data analyzed during this study are included in this article, and further inquiries can be directed to the corresponding author.

## Abbreviations

BC: Breast cancer
WHO: World Health Organization
KSA: Kingdom of Saudi Arabia
TNBC: Triple-negative breast cancer
ACR: American College of Radiology
IRB: Institutional Review Board
ER: Estrogen
PR: Progesterone
HER 2: Human Epidermal Growth Receptor 2
ACR- BI-RADS: American College of Radiology Breast Imaging Reporting and Data System
MRI: Magnetic resonance imaging
NME: Non-mass enhancement
CT: Computed tomography scan
ILC: Infiltrative lobular carcinoma
